# Low Temperature Reactive Sputtering of Thin Aluminum Nitride Films on Metallic Nanocomposites

**DOI:** 10.1371/journal.pone.0133479

**Published:** 2015-07-20

**Authors:** Khaled Sayed Elbadawi Ramadan, Stephane Evoy

**Affiliations:** Department of Electrical and Computer Engineering, University of Alberta, 9211—116^th^ St, Edmonton, Alberta, T6G 2V4, Canada; Gazi University, TURKEY

## Abstract

Piezoelectric aluminum nitride thin films were deposited on aluminum-molybdenum (AlMo) metallic nanocomposites using reactive DC sputtering at room temperature. The effect of sputtering parameters on film properties was assessed. A comparative study between AlN grown on AlMo and pure aluminum showed an equivalent (002) crystallographic texture. The piezoelectric coefficients were measured to be 0.5±0.1 C m^-2^ and 0.9±0.1 C m^-2^, for AlN deposited on Al/0.32Mo and pure Al, respectively. Films grown onto Al/0.32Mo however featured improved surface roughness. Roughness values were measured to be 1.3nm and 5.4 nm for AlN films grown on AlMo and on Al, respectively. In turn, the dielectric constant was measured to be 8.9±0.7 for AlN deposited on Al/0.32Mo seed layer, and 8.7±0.7 for AlN deposited on aluminum; thus, equivalent within experimental error. Compatibility of this room temperature process with the lift-off patterning of the deposited AlN is also reported.

## Introduction

Biological analysis technologies play an important role in many fields such as disease biomarker diagnosis and monitoring, drug discovery, and molecular analysis [1–7]. Established techniques include enzyme-linked immunosorbent assay (ELISA), western blotting, polymerase chain reaction (PCR), and fluorescence conjugated reagents. These assays require labels such as fluorescent dyes and enzymes to properly identify the target. The introduced labels can be toxic to the biological reagents and interfere with the biological or chemical phenomena being investigated. Label-free approaches, on the other hand, keep the biochemical system mostly unperturbed. Biosensors have thus been looked upon as alternative for the monitoring of various biological analytes. Platforms such as quartz crystal microbalance (QCM) [8], micromechanical resonators [9–13], flow cytometry [14, 15], amperometry [16, 17],surface plasmon resonance (SPR) [18–22], magnetoresistance [23, 24], and surface acoustic waves [25] have been considered. The specificity of biosensors is imparted by a probe such as a nucleic acid, an antibody, an enzyme, a cell or an artificial receptor. Different biological probes such as DNA [26], RNA [27], monoclonal [28, 29]and polyclonal antibodies [30], bacteriophages [17, 22, 31–34] and their recombinant binding proteins [22, 35] have been used.

Thin layers of piezoelectric and piezoresistive materials are commonly used to monolithically integrate sensing abilities [36],[37], thus offering numerous advantages over off-chip integration [13, 38]. Aluminum nitride (AlN) is commonly used for such applications. Although its piezoelectric coefficient is an order of magnitude less than ferroelectric materials such as Lead Zirconate Titanate (PZT), AlN offers lower dielectric loss, higher stiffness, lower dielectric constant and higher melting point [39]. Aluminum nitride also enables the realization of high quality ultrathin (<100nm) piezoelectric films without the need of an electrical poling step which is required by PZT [37, 40–42]. In addition, surface acoustic wave (SAW) devices are normally fabricated from piezoelectric materials such as quartz, LiNbO_3_ or LiTaO_3_, or onto thin films such as zinc oxide. These materials are relatively unstable in fluids and offer limited biological compatibility. Aluminum nitride offers better resilience and biological compatibility.

Use of thinner films leads to appreciable performance gains in many applications. For instance, thinner layers increase stress and pressure sensitivity of cantilevers and membranes, as well as the efficiency of energy harvesters. In addition, the performance of resonant mass sensors increases with surface-to-volume ratio, which is readily achieved by reducing the thickness of the structure [36, 43, 44]. On the other hand, static cantilever sensors as well as resonant energy harvesters benefit from higher compliance, which is also increased with reduced thickness [45, 46].

Residual stresses induced by fabrication processes can lead to fracture and device failure. Contact metals used as electrodes in piezoelectric devices should thus possess high enough tensile strength to withstand these stresses. To such effect, novel co-sputtered metal nanocomposites optimized for the fabrication of micromechanical devices have been reported [47–49]. These nanocomposites feature grain size as small as a few nanometers and residual stresses substantially lower than those of pure metals. Also, control of co-sputtering parameters facilitates precise control of residual stress thus enabling microcantilevers with high surface area and ultrathin thicknesses. More specifically, a metal nanocomposite composed of 68 atomic percent (at. %) Al and 32 at. % Mo has been described [48, 50]. The high strength and low surface roughness of the material allowed the realization of cantilevers as thin as 4.3 nm. More recently, the fabrication of conductive nanocomposite AlMo membranes as thin as 28 nm and high fracture strength has also been reported [51]. The properties of the Al/0.32Mo (i.e. at. 32% Mo) are additionally promising for the realization of ultra-thin AlN/AlMo piezoelectric bimorph structures. We have examined the use of such metallic glasses as a seed layer for the growth of AlN thin films. Such an understanding is of importance for the eventual realization of any devices that would leverage the features of such a piezoelectric/metallic glass bilayer.

The piezoelectric properties of AlN film is usually related to growth in its (002) orientation [52]. Prior studies showed that for a given sputtering system, an optimal combination of sputtering power and pressure exists for the growth of AlN thin films with preferential c-axis orientation [53]. These optimal growth parameters may however vary from system to system. Higher deposition temperature also enhances c-axis growth by increasing the kinetic energy and surface mobility of the deposited aluminum atoms [53, 54]. It, however, limits the patterning options of the AlN thin film. Higher deposition temperature also induces undesirable stress in the grown thin films due to thermal expansion mismatch between AlN and the seed material. The nature of the seed material on which AlN is deposited plays an important role in the film quality. In addition to thermal expansion mismatch, a differing crystal orientation of the substrate has been known to inhibit the desired c-axis orientation growth. More specifically, previous work showed that (111) oriented seed layers are more likely to lead to higher c-axis or (002) orientation of the grown AlN film [55–57]. This can be observed for platinum, aluminum and silicon (111) compared to silicon (100). For example, Caliendo *et al*.[55] reported a 2.5 times higher ratio between the x-ray diffraction (XRD) peak intensities of AlN (002) and (101) when Si (111) instead of Si (100) was used as a seed material. Also, Singh *et al.* [56] confirmed the highest (002) crystallinity of AlN was obtained when using Al (111) as seed, compared to Si (100), SiO_2_ and gold. Jin *et al.* [57] also reported that the surface roughness of the seed also affects the AlN c-axis orientation growth. Reducing the Al seed layer thickness reduced its surface roughness and yielded better AlN c-axis crystallinity. Studies of (002) crystallinity variation based on the thickness of the AlN layer grown are more important when it comes to piezoelectric NEMS. Galca *et al*.[58] reported that the (002) crystallinity of AlN grown on Si (100) increases with film thickness. They also observed an amorphous AlN-Si interface layer of 5nm thickness, and no significant preferential (002) peak in films thinner than 30nm. There is thus yet to be any report of NEMS devices involving an AlN piezoelectric layer thinner than 50nm [37, 40–42].

Seed layer surface roughness and crystal orientation are thus known to affect the AlN thin film quality. In turn, Ophus *et al*.[48] showed that the surface roughness of sputtered Al/0.32Mo is two orders of magnitude smoother than pure aluminum for a film thickness of ~1.5μm. The crystalline morphology of such films is however more amorphous-like than poly-crystalline, potentially offsetting the possible gains from the lower surface roughness. Pure aluminum thin films with low surface roughness could alternatively be obtained by evaporation. However, the weak mechanical strength of pure aluminum remains an unavoidable disadvantage for nanofabrication. Given the potential of AlN/metallic glasses bilayers for the realization of ultra-thin bimorphs, an assessment of AlN film quality realized onto such metallic nanocomposites is thus needed. Finally, the AlN films reported in this study were also deposited at a relatively low temperature. This reduces the residual stress and also allows more flexibility in the fabrication process due to selectivity issues between AlN, Al and Al/0.32Mo etchants. This room temperature deposition process also allowed the use of a lift-off process to pattern the aluminum nitride, as most photoresists would otherwise degrade at high temperature deposition. This report therefore introduces the possibility of thin AlN/metallic glass bilayers for applications in sensors and energy harvesters. In addition, the films are realized using a low-temperature deposition process offering enhanced compatibility with other fabrication techniques.

## Methods

Ten samples of AlN films were grown with different deposition parameters in order to analyze the impact of such parameters on film properties. Table 1 summarizes the deposition parameters of each sample. A seed layer of Al/0.32Mo glassy nanocomposite was first deposited by co-sputtering on piranha cleaned silicon wafers, using previously reported deposition techniques [50]. The sputtering system was equipped with 3 magnetron targets, and the target-to-substrate spacing was fixed at 6 cm. A cryogenic pump was used to get to the high vacuum pressure necessary for the system. The flow of argon and nitrogen was controlled with gas flow sensors, and the targets were connected to DC power supplies. Pulsed DC [59] and radio frequency power [54] have also been reported. Use of such power sources however fell outside the scope of this study. DC reactive sputtering of AlN has been previously reported by different research groups [60, 61]. In our case, we never encountered target poisoning effects when growing AlN using a DC power supply. For depositing AlN, two aluminum targets of 99.999% purity were used in order to increase the deposition rate. We first determined reasonable sputtering parameters for high c-axis preferentially oriented AlN on Al/0.32Mo. We then deposited AlN on Al seed layer using parameters for comparison purposes. In the first four samples, the sputtering power was fixed at 300W while the pressure was changed from 1–4 mTorr (samples M1, M2, M23 and M3). One deposition run at 300W and 1mTorr was carried at a higher substrate temperature of 250°C in order to assess effect of temperature on the deposited film (sample M11). The sputtering power was then changed from 200W to 50W while fixing the pressure at 1mTorr (samples M14, M24 and M15).

**Table 1:** Deposition parameters for the different samples presented in this study. The symbols *P*, *p*, *T*, *τ*, *t* stand for sputtering power, sputtering pressure, substrate temperature, deposition time and thickness, respectively. RT stands for room temperature.

Sample	Seed Material	Seed Thickness (nm)	*P*(W)	*p* (mTorr)	*T*(°C)	*τ*(s)	*t* (nm)
M1	Al/0.32Mo	130±8	300	1	RT	2520	470
M2	Al/0.32Mo	130±8	300	2	RT	2520	475
M23	Al/0.32Mo	130±8	300	3	RT	3700	453
M3	Al/0.32Mo	130±8	300	4	RT	2280	446
M11	Al/0.32Mo	130±8	300	1	250	2460	450
M14	Al/0.32Mo	130±8	200	1	RT	4380	464
M24	Al/0.32Mo	130±8	100	1	RT	9000	450
M15	Al/0.32Mo	130±8	50	1	RT	7800	200
M16	Al (111)	120±10	200	1	RT	5400	518
M17	Al (111)	120±10 nm	300	1	RT	2520	480

Reactive sputtering of AlN was then carried out on pure Al at 200–300W powers and 1mTorr pressure (samples M16 and M17). As discussed below, a pressure of 1 mTorr was employed for those samples as it represented best compromise between relative (002) XRD peak intensity and film stress. A 2:1 Ar/N_2_ flow ratio was employed in all experiments. All AlN films reported had thicknesses of 470nm±23nm, except for sample M15 which employed a lower pressure and thus a significantly lower deposition rate (Table 1). The base pressure was kept slightly below 10^-6^Torr. No significant changes in the resulting films were observed when deposition was performed at lower pressures (ie between 10^−6^ and 10^−7^ Torr).

**Fig 1:**
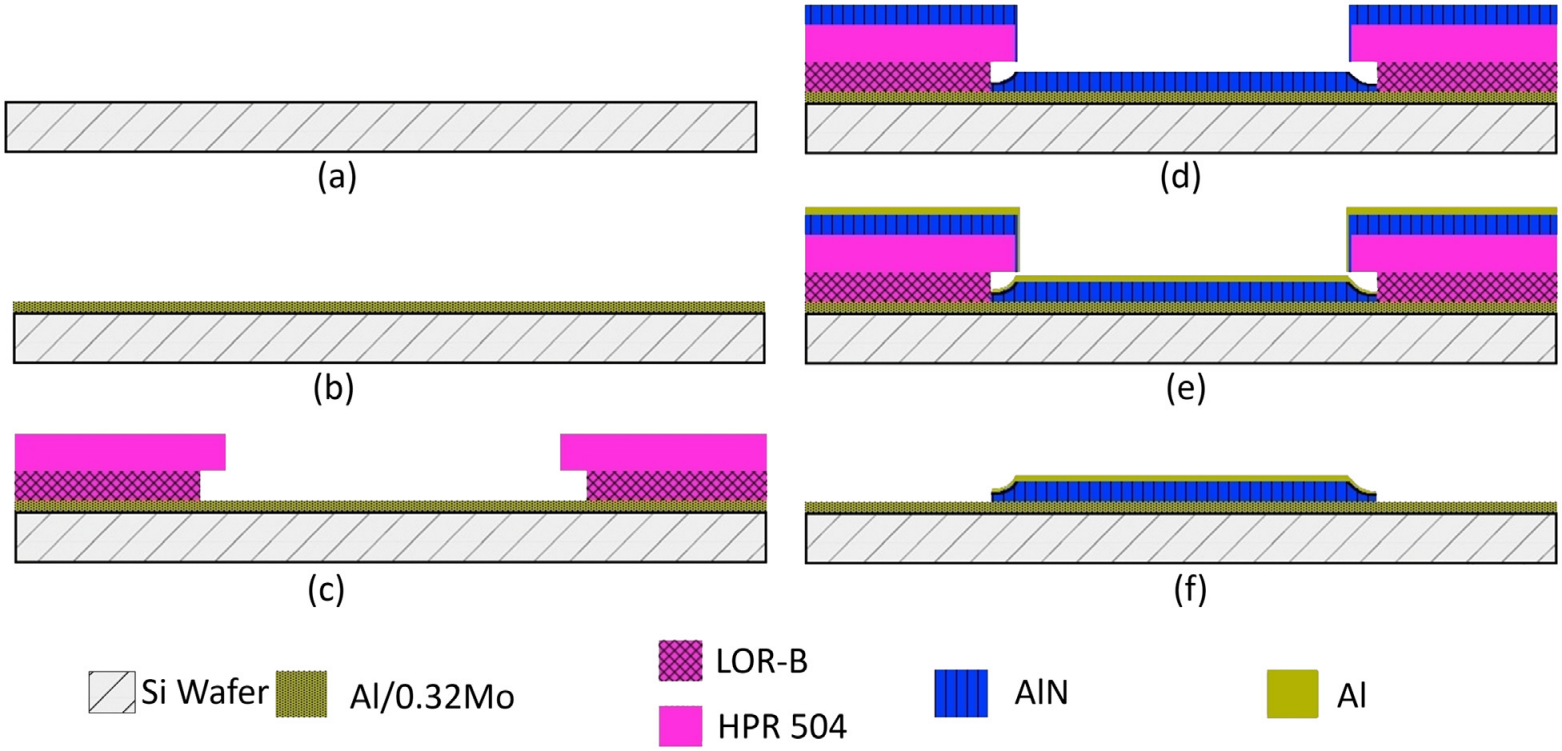
(Color Online) Lift-off process for aluminum nitride two plate capacitors. (a) Piranha cleaned Si wafer, (b) Co-sputtering of Al/0.32Mo nanocomposite, (c) Photolithography process for patterning a bi-layer of LOR-B and HPR 504 resists, (d) Reactive DC sputtering of AlN, (e) Sputtering of Al top electrode and (f) Stripping the photoresist to define the capacitors.

Performing reactive sputtering of AlN at room temperature enables the patterning of the deposited film using a resist lift-off process. Higher deposition temperature would otherwise degas any resist present on substrate, contaminate the deposited AlN thin film, and degrade resulting crystallinity. As alternative to lift-off, patterning by selective etching would require wet chemicals such as tetra methyl ammonium hydroxide (TMAH) and phosphoric acid based solutions (PWS), or dry etchants such as using chlorine or fluorine based plasmas [52]. Poor etch selectivity between AlN, Al/0.32Mo and Al however precludes using such methods. In return, a lift-off process leveraging photoresists that can withstand temperature of up to 100°C is implemented with the low-temperature AlN deposition process (Fig 1). In addition to the ten samples mentioned above, 4 samples were prepared with this fabrication process including AlN layers deposited on two different seed layers (Al and AlMo) and two different sputtering powers (200W and 300W). This process was used to realize parallel plate capacitors using AlN as dielectric. The thicknesses were150nm, 2μm, 1.5μm, 500nm and 150nm for the Al/0.32Mo bottom electrode, LOR-B resist layer, HPR-504 resist layer, AlN layer and Al top electrode layer respectively. The process starts with piranha cleaning of the Si (100) wafers. The Al/0.32Mo films were then deposited by co-sputtering at 5mTorr. The powers to the aluminum and molybdenum targets are calibrated to match the 32% atomic percentage of Mo needed. Thirdly, a standard optical lithography process is carried out using a by-layer resist of LOR5B and HPR-504. The AlN and Al layers are then deposited onto the patterned resist. The lift-off process is completed by stripping the bilayer resist in an ultrasonic bath of Remover PG. The dielectric properties of AlN were extracted by characterizing these parallel plate capacitors using a Keithley 4200-SCS parameter analyzer. This fabrication process enables feature sizes as small as 3–4μm. Other techniques such as electron beam lithography could potentially be used with thinner layers and enable nanoscale features [13].

A stress measurement system (Flexus 2320, Tencor, USA) was used in order to study the biaxial stress of the deposited AlN films. This system uses an optical method to measure the curvature of the wafer and accordingly calculates the residual stress of the deposited film. X-ray diffraction (XRD) was employed to characterize the crystallinity of the films. The XRD instrument (Ultima IV, Rigaku, Japan) employs a Cu-*K*_α_ radiation (λ = 0.154nm) working at 40kV and 44mA. The XRD spectra were obtained at scanned angle 2θ varying from 30°–70°. Three measurement runs were carried out at different locations of each sample. Rocking curve XRD measurements were also carried out on a single location of each sample using a different XRD instrument (D8 Discover, Bruker, USA) to gauge the quality of the c-axis crystallization. Rocking curve XRD measurements were performed by fixing the 2θ at the (002) peak position and rocking the sample across the ω axis. Biaxial stress was also assessed from the XRD data using a standard methodology, providing supplemental assessment to the stress measurement [60]. In this approach, the c-axis strain is calculated from ε_z_ = (c-c_o_)/c_o_, where c_o_ is the strain-free lattice constant (4.975 Å) and c is the lattice constant of the deposited film as measured from the position of the (002) peak. The biaxial stress σ can be calculated from σ = [(C_13_–(C_11_+C_12_)(C_33_/2C_13_)] ε_z_, where C_ij_ values correspond to the theoretical stiffness tensor of AlN, namely C_11_ = 396GPa, C_12_ = 137GPa, C_13_ = 108GPa, and C_33_ = 373GPa [62].

Atomic force microscopy (AFM) (MFP-3D, Assylum Research, USA) was used to study the impact of seed layer surface roughness on the crystallinity of the sputtered AlN thin film. The AFM data was processed using the Scanning Probe Image Processor (SPIP v. 6.2.0) software. Field emission scanning electron microscopy (SEM) (Sigma, Zeiss, Germany) was used to investigate the columnar growth of the AlN thin film and the quality of the lift-off process.

The piezoelectric response of the AlN films deposited at optimized processing parameters was carried out. We characterized the *e*_*31*, *f*_ transverse piezoelectric coefficient using a common beam vibration method, following the procedure reported by Jaber *et al*.[63]

## Results and Discussion

**Fig 2:**
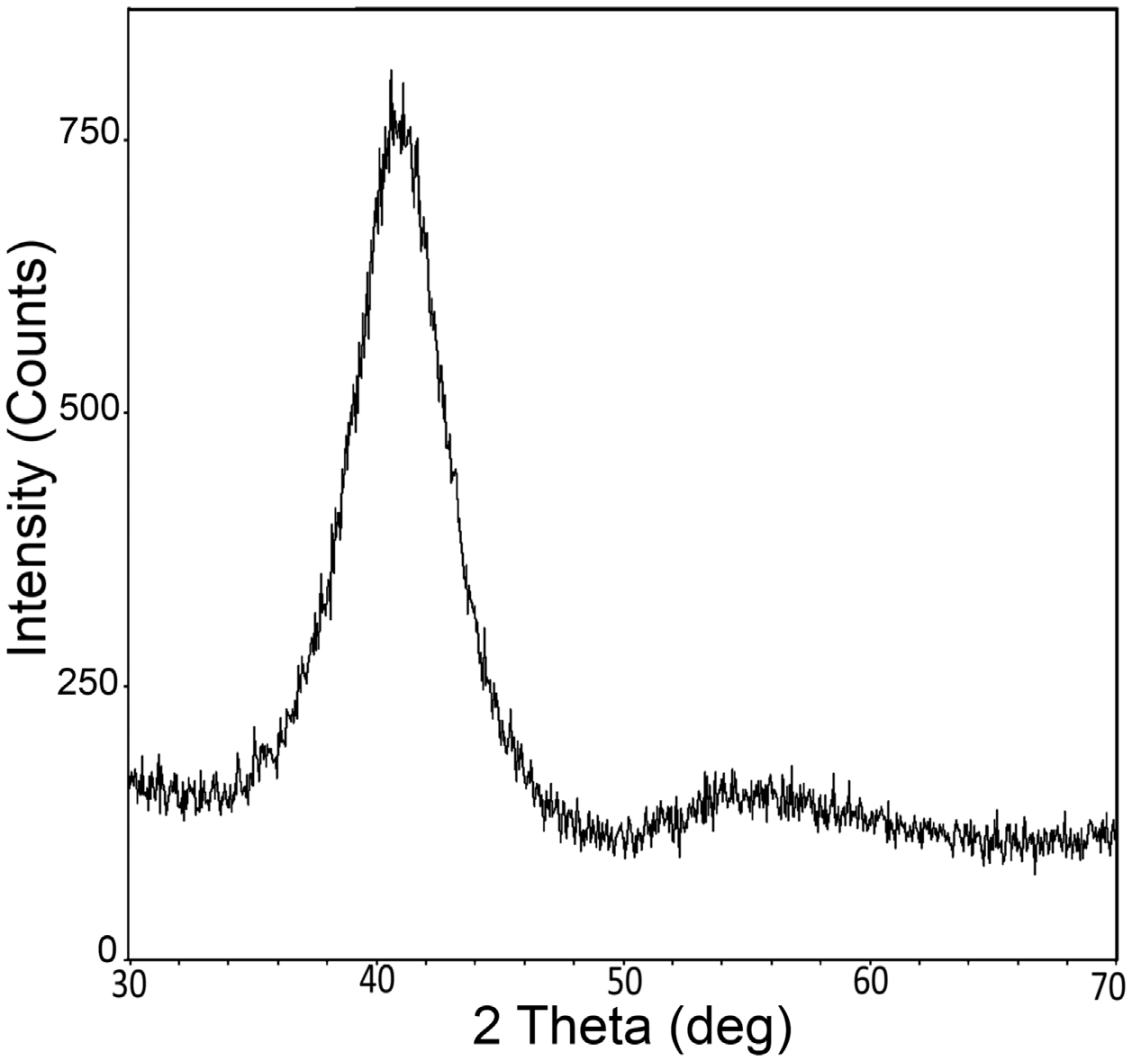
X-ray diffraction spectrum of Al/0.32Mo nanocomposite.

**Fig 3:**
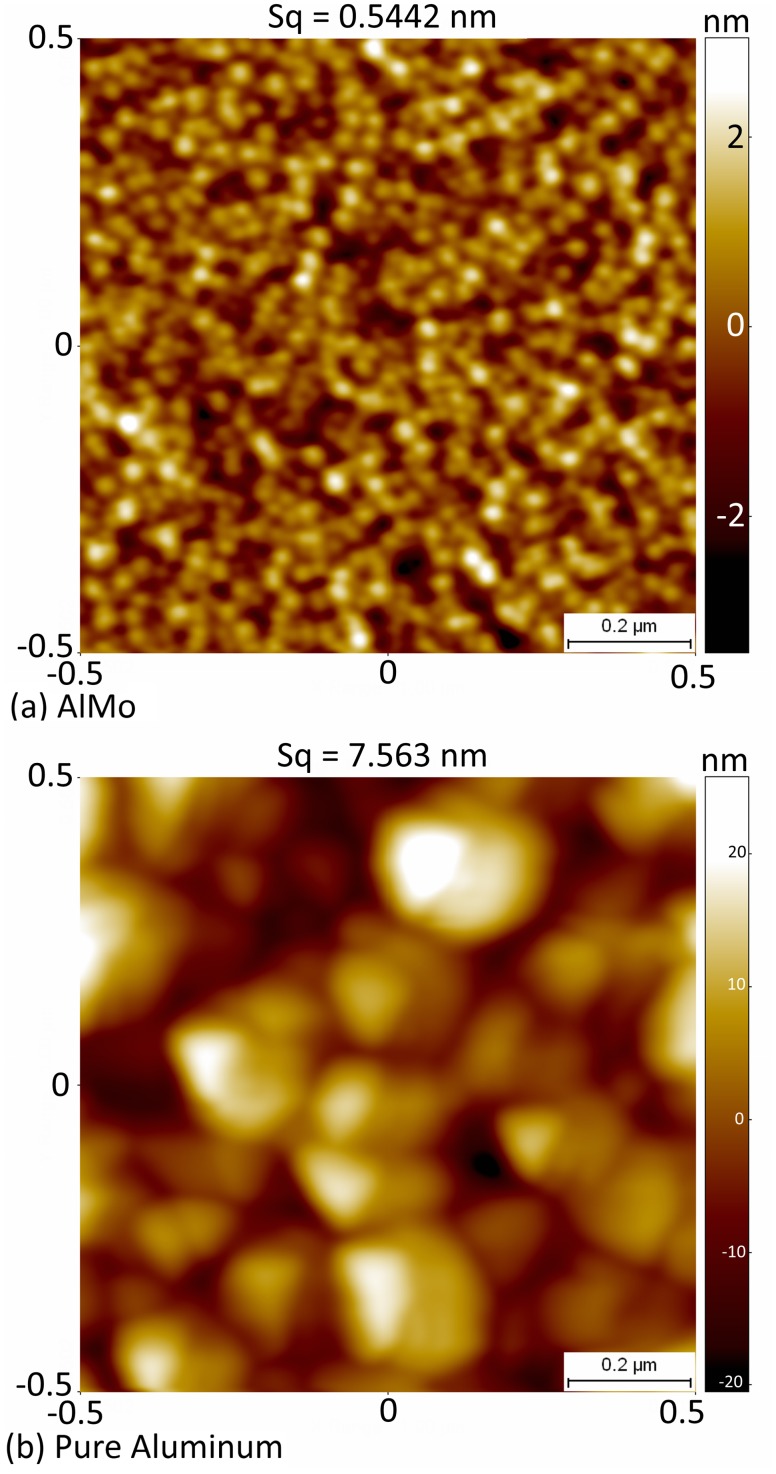
(Color Online) AFM surface images of 1μm×1μm area for: (a) the Al/0.32Mo nanocomposite thin film with 130nm thickness and (b) the Aluminum thin film with 120nm thickness. Sq is defined as the root mean square roughness, which is calculated using Scanning Probe Imaging Processor SPIP software version 6.2.0.

Understanding of the structural differences between the Al/0.32Mo nanocomposite and pure aluminum is critical in order to assess possible effect on AlN growth. Fig 2 shows the broad XRD peak of Al/0.32Mo at 2θ = 41° in addition to other minor broad peaks, which is a signature of AlMo at the 0.32 composition. This broad band is indicative of the amorphous-like nature of the Al/0.32Mo [48] and will overlay the main (002) and (101) peaks of AlN. Atomic force microscopy was used to investigate the surface of both seed layers (Fig 3). Those images confirm that the roughness of the pure aluminum is an order of magnitude higher than that of the Al/0.32Mo nanocomposite. The difference between our measured roughness and previously reported values [48] is attributed to the use of thinner films (~130nm) compared to the film thickness of ~1.5μm studied by Ophus *et al*.[48] The surface roughness of pure aluminum indeed increases with higher film thickness. Use of Al/0.32Mo metallic glasses for such a higher film thickness therefore showed a *two* orders magnitude improvement of smoothness over pure aluminum, compared to the *one* order of magnitude improvement observed here. Fig 3 also shows the smaller grain size of the Al/0.32Mo nanocomposite compared to that of pure aluminum. These two structural characteristics (nanocrystalline-amorphous phase and lower surface roughness) are expected to affect the properties of AlN grown on such seeds.

The resistivity of Al/0.32Mo nanocomposite was measured to be 5.9±0.3 μΩ-m using a 4 point probe approach. This value is two orders of magnitude higher than the resistivity of sputtered pure aluminum thin films, which was measured to be 0.079±0.002 μΩ-m. The higher resistivity of Al/0.32Mo is the result of its smaller grain size and amorphous phase compared to pure aluminum [50]. For the mechanical strength, Lee *et al*.[50] reported nanoindentation hardnesses of 6.3GPa and 0.6GPa for Al/032Mo and pure aluminum, respectively. In spite of the higher resistivity, the mechanical strength of Al/0.32Mo over pure Al provides important advantages for the realization of ultra-thin and/or ultra-narrow mechanical structures.

**Fig 4:**
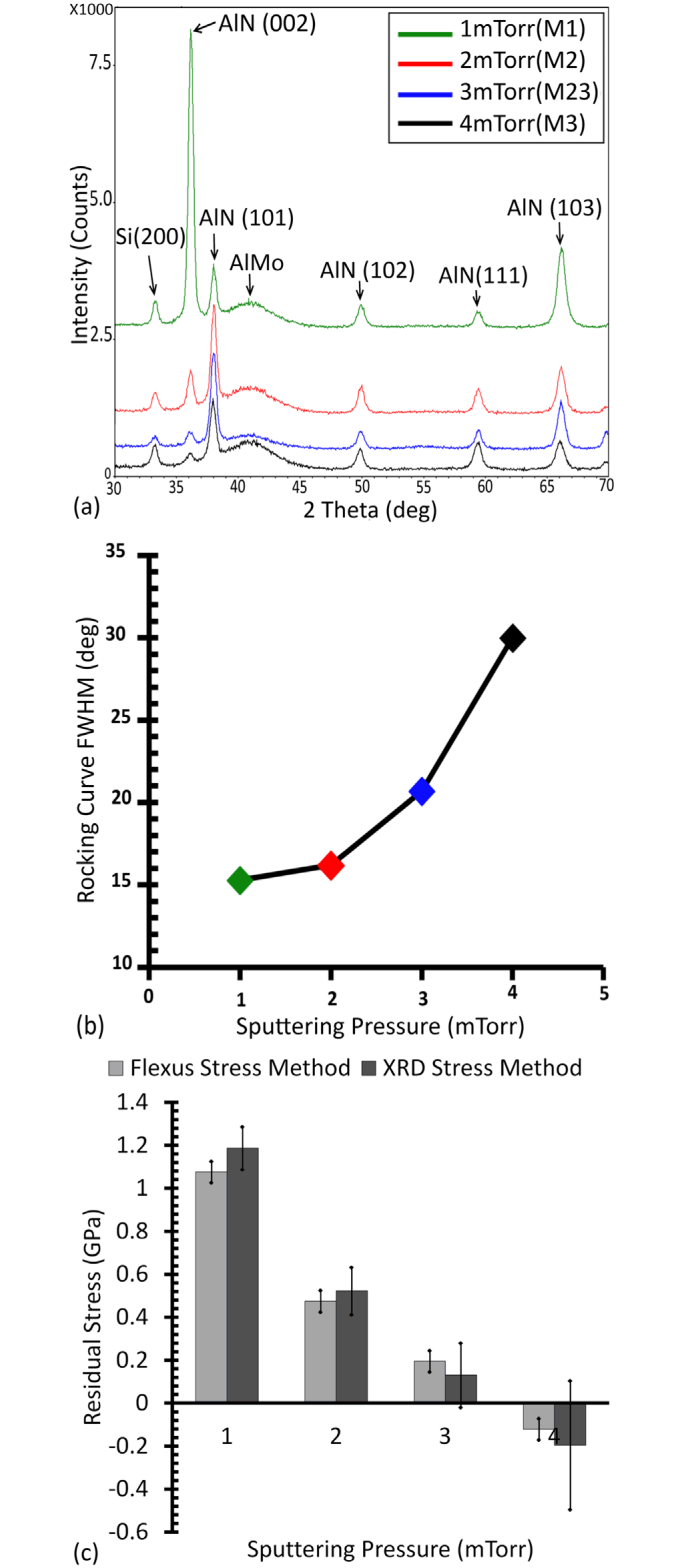
(Color Online) Sputtering pressure impact on AlN grown on AlMo. (a) shows the x-ray diffraction spectra of deposited AlN thin films at room temperature, a power of 300W and sputtering pressure of 1–4 mTorr (Sample name is included in the legend). (b) and (c) show the FWHM of the rocking curve and the AlN residual stress as a function of sputtering pressure, respectively. The line between the data points in (b) is meant to guide the eye.

Fig 4a shows typical XRD spectra for AlN deposited at 300W and pressures of 1, 2, 3 and 4 mTorr. The XRD spectra of samples M1, M2, M23 and M3 suggest that the (002) crystallographic texture increases with decrease of sputtering pressure. The FWHM of the rocking curve indeed decreased with decreasing sputtering pressure (Fig 4b), as previously reported [52, 56, 60]. This trend is explained by the higher mean free path of the aluminum atoms ejected from the target at lower sputtering pressures. Xu *et al*.[61] suggest that the (002) orientation will be increasingly preferred if this mean free path is greater than the distance between the target and the substrate. Aluminum atoms would indeed be reaching the substrates with higher energies, facilitating the formation of Al-N bond in the (002) direction [61].

In return, the resulting thin films show higher residual stresses, which is undesirable. This can be seen in Fig 4c where the residual tensile stress increases with decreasing pressure. The results show a close matching between the XRD stress calculation method and the Flexus stress measurement. The error bars in the Flexus stress measurement values represent the standard deviation of the stress based on the thickness variation of the AlN layer across the wafer. The standard deviation in the XRD stress measurement is based on the difference between the three different locations measured on each sample. The relatively weak (002) peak obtained from film deposited at 4 mTorr resulted in a higher deviation of XRD stress measurement calculation. The Flexus tool is thus deemed to be more reliable for the measurement of stress in such conditions. The stress of the films deposited at 1mTorr is indeed higher than those deposited at 2mTorr. However, the relative (002) peak intensity is significantly higher in films deposited at 1mTorr. This deposition pressure was thus selected based on this figure of merit, and said residual stress somewhat mitigated by lowering the deposition power. For instance, we did perform depositions at 2mTorr using powers of 400W and 200W. Unfortunately, the relative peak intensities of those films were in the range of 20–40% while the ones on 1mTorr were in the range of 60–80%. Use of a pressure of 1 mTorr in combination with lower power was thus deemed as optimal approach to obtain the best compromise between (002) crystallinity and film stress.

**Fig 5:**
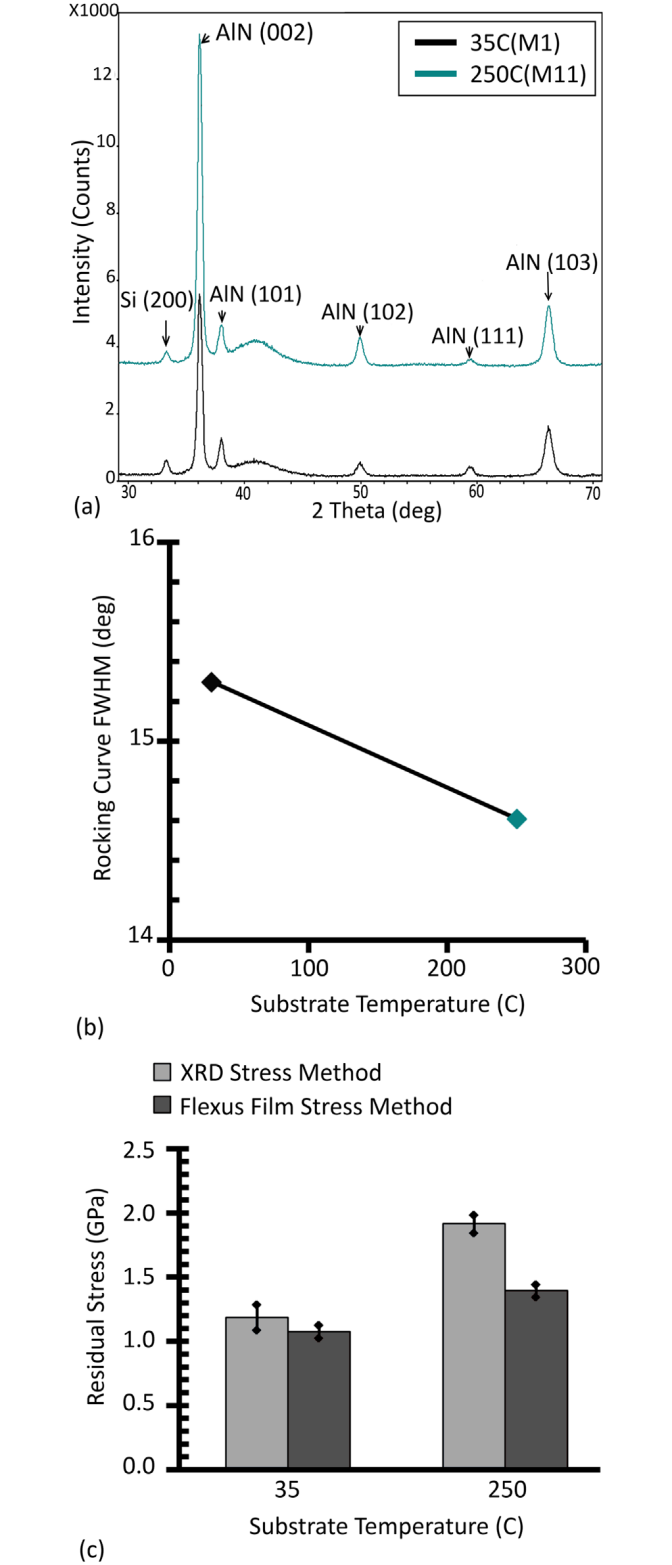
(Color Online) Substrate temperature impact on AlN grown on AlMo. (a) shows X-ray diffraction spectra of deposited AlN thin films at a power of 300W, a pressure of 1mTorr and two different substrate temperatures. (b) and (c) show the FWHM of the rocking curve and the AlN residual stress as a function of substrate temperature, respectively.

Effect of substrate temperature on AlN growth has been previously studied [64]. Here, a single deposition run at 250°C was carried out at a pressure (1mTorr) to verify impact of deposition temperature. Use of higher temperature did improve the (002) growth of AlN, as indicated by the higher intensity of the (002) peak compared to the other minor peaks (Fig 5a). The variation in FWHM of the rocking curve from 15.3° to 14.6° (Fig 5b) is however not significant compared to the difference observed when changing sputtering pressure or power. Use of high temperature however increases the film stress (Fig 5c). As mentioned previously, it would also preclude the use of a lift-off process for the patterning of the deposited films.

**Fig 6:**
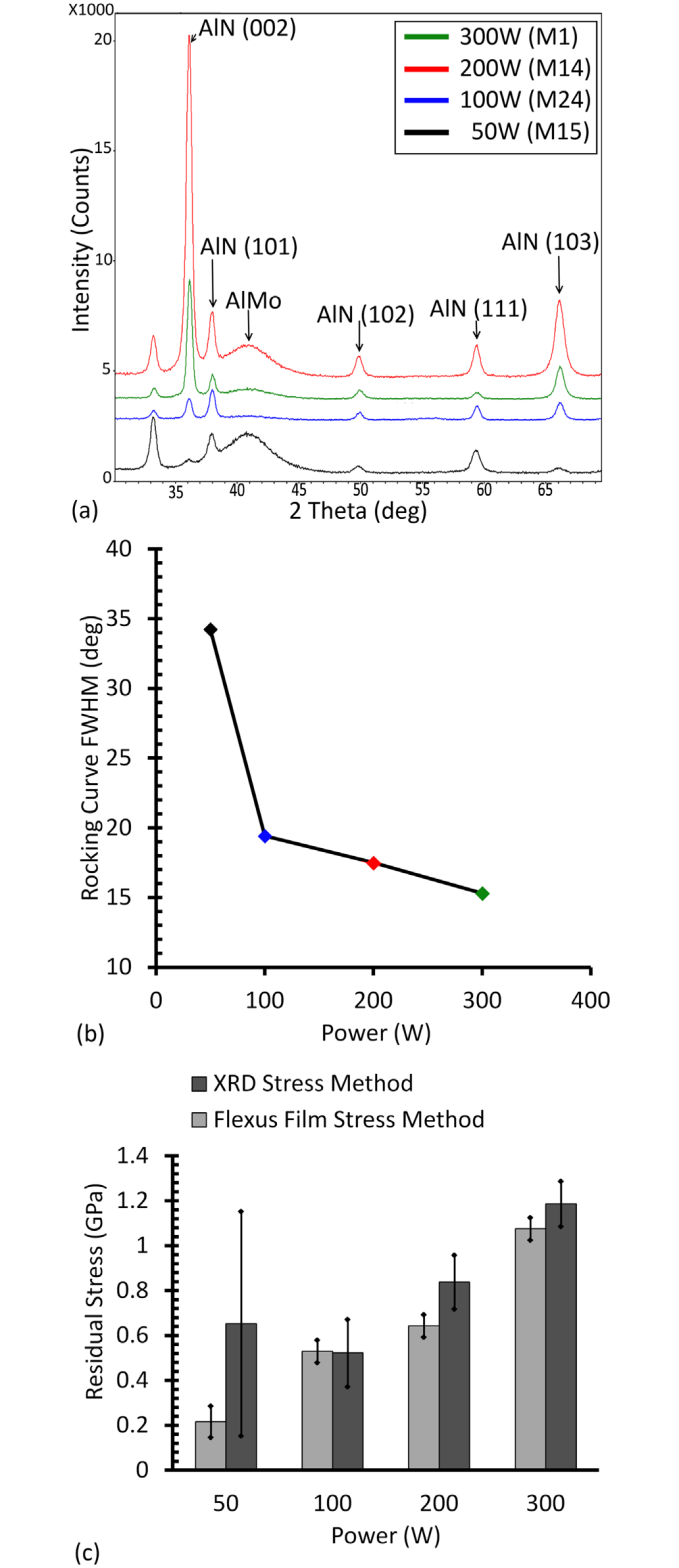
(Color Online) Sputtering power impact on AlN grown on AlMo. (a) shows X-ray diffraction spectra of deposited AlN thin films at room temperature, a sputtering pressure of 1mTorr and power of 50–300W. (b) and (c) show the FWHM of the rocking curve and the AlN residual stress as a function of sputtering power, respectively.

To optimize the residual stress, AlN thin films were prepared at a sputtering pressure of 1mTorr and four different sputtering powers of 50W, 100W, 200W and 300W, respectively. Fig 6 reports the c-axis quality and the residual stress of each film. Growth in the (002) direction is completely inhibited in favor of the (101) one at powers below 100W (Fig 6a). The mean free path of the ejected Al atoms remains greater than the substrate to target distance. As such, there should still be very low chance of atom collision at these pressures. The reduced (002) crystallographic orientation may alternatively be the result of the low power not supplying enough kinetic energy for the adsorbed atoms to properly rearrange in the (002) orientation [24]. Although the (002) peak intensity showed its highest value for AlN film deposited at 200W (Fig 6a), the FWHM of the rocking curve maintained the decreasing trend with increasing sputtering power (Fig 6b). The deviation in residual stress is very significant at the 50W sputtering power compared to higher powers, as indicated Fig 6c. Similar to the films deposited at a 4mTorr pressure, the low peak of (002) renders the measurement unreliable. The Flexus system thus becomes a more reliable tool for such stress assessment.

**Fig 7:**
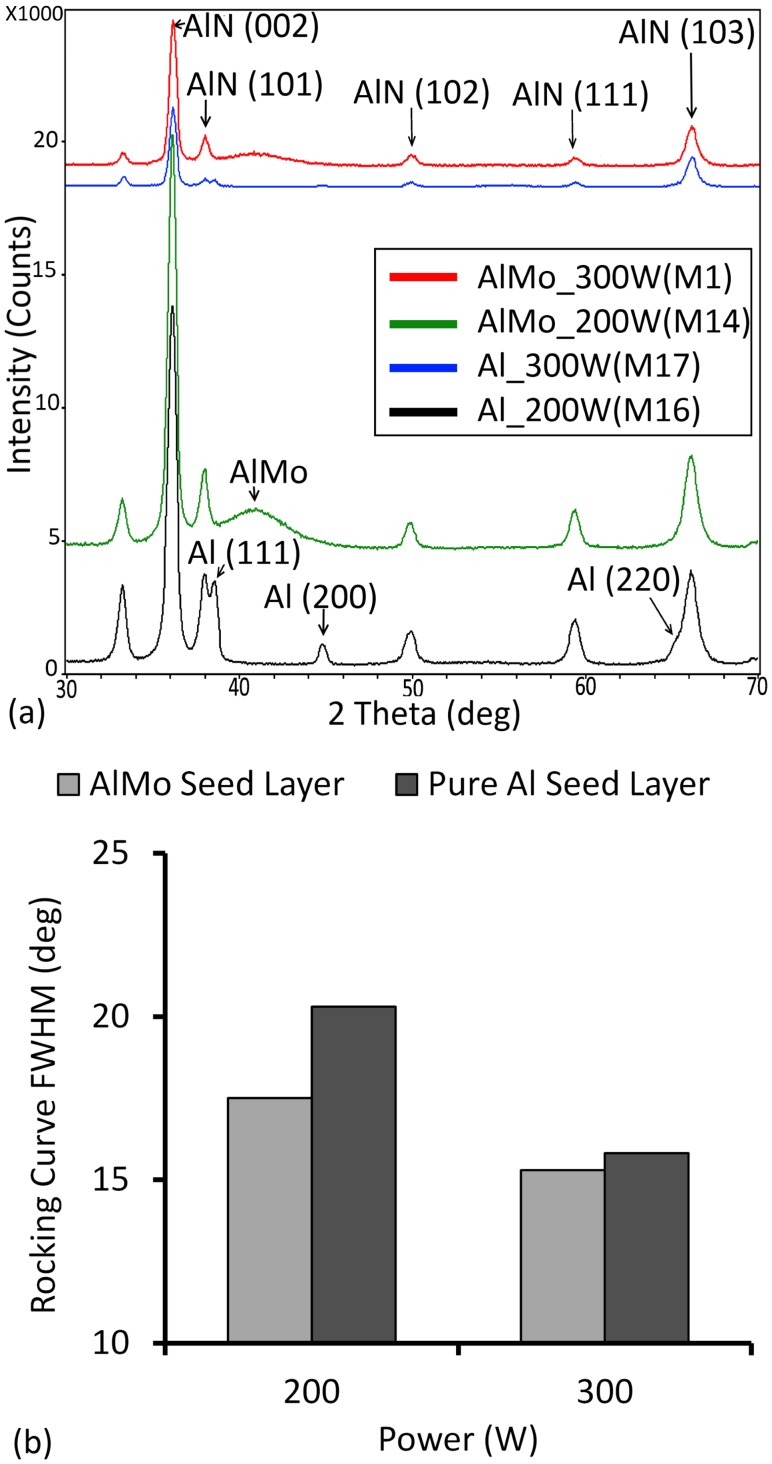
(Color Online) Seed layer impact on AlN. (a) shows the X-ray diffraction spectra of AlN thin films grown on AlMo at 200W (M14) and 300W (M1) and, on pure aluminum at 200W (M16) and 300W (M17). (b) shows the FWHM of the rocking curve for the four samples as a function of power and seed layer.

Based on these findings, the comparison between Al/0.32Mo and aluminum as seed layers for c-axis AlN growth has been carried out at 1mTorr pressure and sputtering powers of 200W and 300W, respectively. AlN films were thus also deposited on aluminum thin films of 120nm±10nm thickness. Results shown in Fig 7a suggest that the intensity of the (002) peak for AlN on Al/0.32Mo is higher than thin films deposited on aluminum at both 200W and 300W powers. However, the FWHM of the rocking curve are however equivalent within experimental error for both films grown on Al/0.32Mo and Al, respectively. These findings suggest that c-axis oriented AlN can be deposited on the nanocrystalline-amorphous Al/0.32Mo films with a quality somewhat similar to films deposited on aluminum. As indicated previously, the surface of Al/0.32Mo is much smoother than that of pure aluminum. On the other hand, the amorphous-like nature of those films could counter any gain obtained from the smoother surfaces. This trade-off between improved roughness and reduced crystallinity apparently results in AlN films of comparable crystallographic properties. This finding is supported by a study of the impact of aluminum seed layer thickness on the growth of AlN films [57]. This study found that as the thickness of the Al layer decreases, the crystallinity increases for the same sputtering parameters. The authors explained the trend through the decrease of Al surface roughness obtained as a result of decreasing the Al seed layer thickness. This being said, the higher structural strength of AlMo compared to pure Al however offers potent prospects for the realization of ultra-thin and/or ultra-narrow bimorphs.

**Fig 8:**
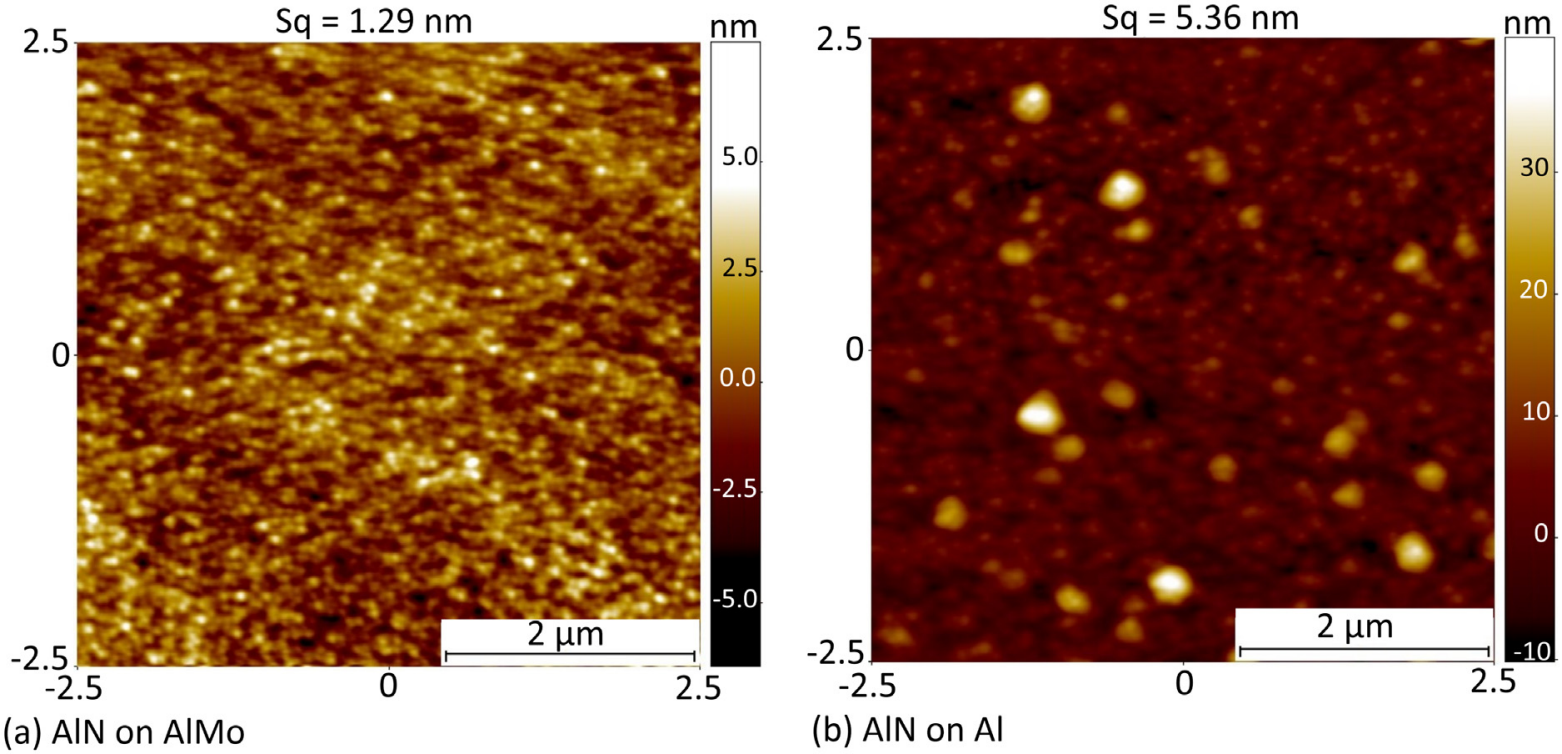
(Color Online) AFM Surface Images of 5μm×5μm area for: (a) AlN grown on AlMo nanocomposite (M14), and (b) AlN grown on pure Al (M16). Both layers are deposited with the same sputtering conditions of 200W power and 1mTorr pressure.

**Fig 9:**
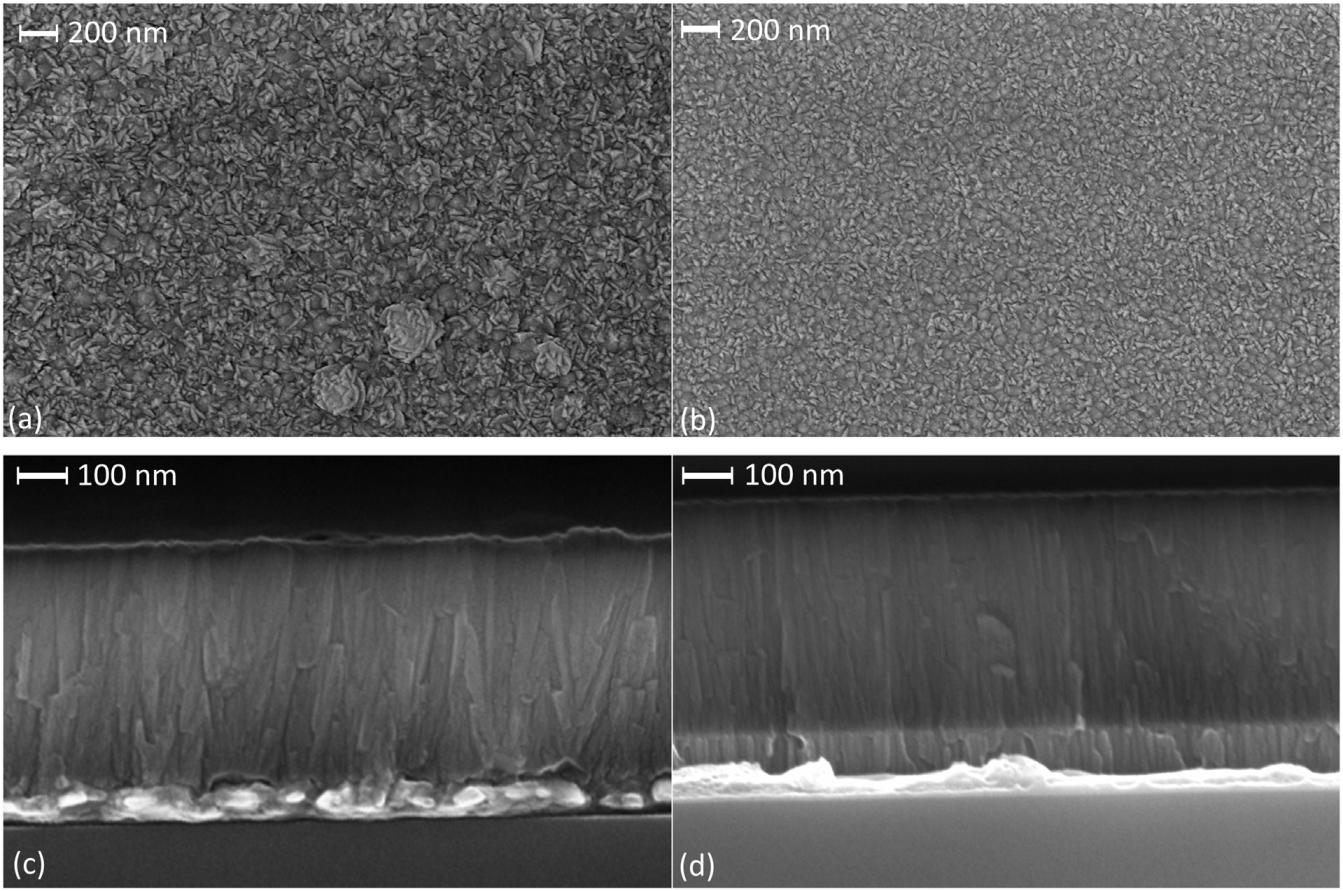
SEM images of AlN thin films grown on different seed layers with DC reactive sputtering at 200W power and 1mTorr pressure. surface (a) and cross-section (c) of AlN grown on pure Al (M16), and surface (b) and cross section (d) of AlN grown on AlMo nanocomposite (M14).

In addition, the relative roughness of the seed layers employed was also reflected in the resulting smoothness of the deposited AlN films (Fig 8). The AFM images shown in Fig 8 were obtained on films deposited using a deposition process of 200W power and 1mTorr pressure. For an area of 5μm×5μm, a homogeneous grain size of 70–100nm is observed for the AlN grown on the smoother Al/0.32Mo nanocomposite, yielding a surface roughness of 1.29nm. On the other hand, AlN films grown on pure aluminum showa higher roughness of 5.36nm. High resolution field emission SEM imaging supported the AFM findings. Fig 9 shows such images of surfaces and cross-sections of AlN films grown on Al/0.32Mo and Al. In the case of the Al seed layer, repeated rough circular grains were observed ranging from 100nm to 500nm in size. On the other hand, the size of columnar grains is consistently less than 100nm when Al/0.32Mo was used as seed layer. The cross section image confirms the presence of these larger grown columns in the films grown on pure Al with more rounded top surface. The use of Al/0.32Mo as a seed layer, therefore, reduces the AlN surface roughness, which affects the performance of piezoelectric surface acoustic wave devices.[65]

**Fig 10:**
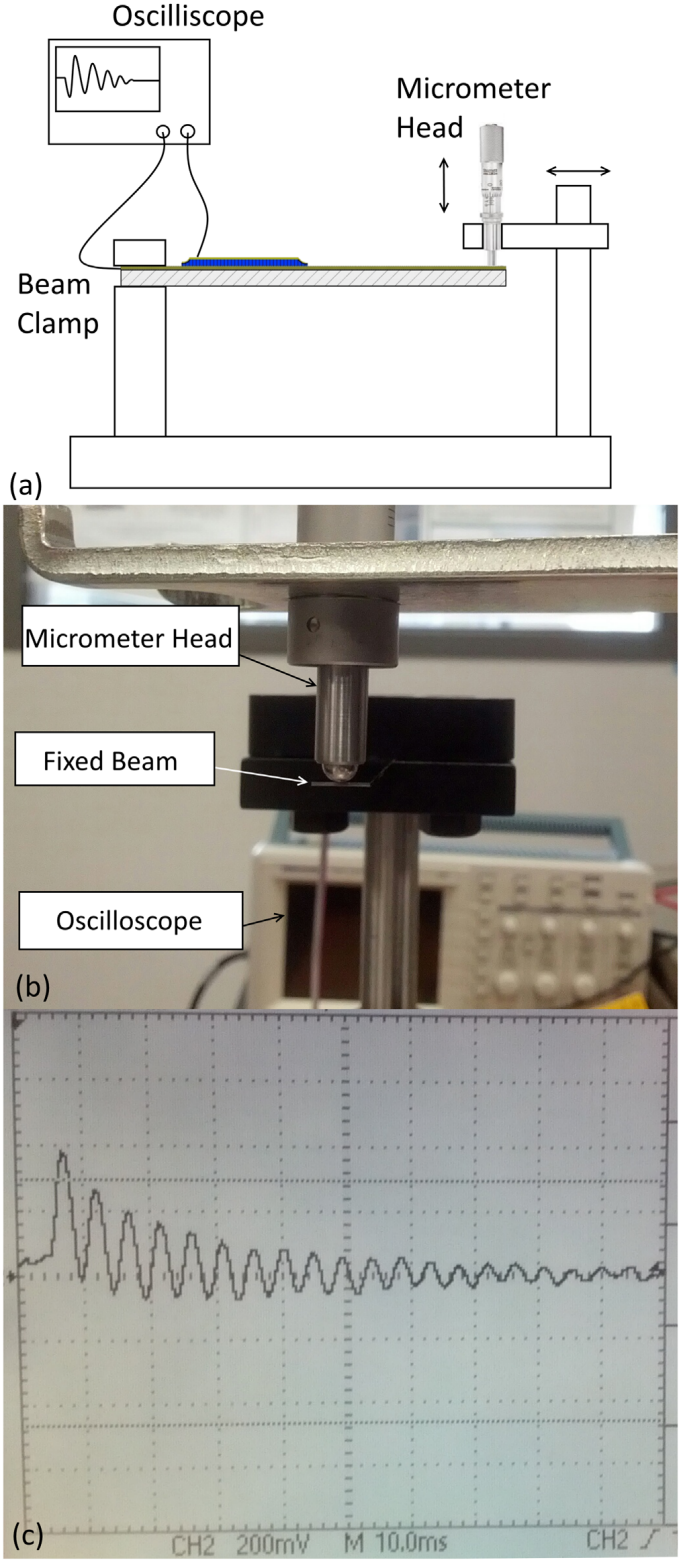
Piezoelectric characterization of AlN. (a) schematic of the testing setup, (b) shows a fabricated silicon beam with piezoelectric AlN (the shaded area in the beam is fixed and the deflection is applied in the direction of the arrow depicted), (c) shows the piezoelectric characterization setup and (c) shows the voltage response of the beam at a deflection of 1 mm.

The *e*_*31*, *f*_ transverse piezoelectric coefficient of AlN deposited on both pure Al and Al/0.32Mo were also assessed using the common beam vibration method (Fig 10a)[63]. Fig 10b shows a representative sample of beams fabricated from piezoelectric AlN layers. The AlN films were 1.2 μm thick in both cases, and were deposited at room-temperature. An end-point displacement of 1 mm was applied and the voltage response recorded using an oscilloscope. Fig 10c shows the actual test setup while Fig 10d shows a typical electrical signal observed. The piezoelectric coefficients were found to be 0.46±0.1 C m^-2^ and 0.9±0.1 C m^-2^, for 1.2μm thick AlN deposited on Al/0.32Mo and Al, respectively. In comparison, the piezoelectric coefficient of 1μm thick AlN sputter-deposited on Pt at T = 400°C has been reported at1.05±0.05 Cm^-2^ [52].

In summary, use of a metallic glass as a seed layer yielded the deposition of AlN of quality and performance that is comparable to films deposited on pure Al. A slight decrease of piezoelectric response could be attributed to a slightly reduced crystallinity of the deposited film, which is expected given the amorphous nature of the seed layer. Nonetheless, the piezoelectric response remains within range of values reported in literature [18,19]. In return, use of metallic glass as a seed layer yielded deposited films of improved smoothness, an important figure of merit for many applications involving surface waves and/or requiring ultra-thin layers. The impact of such improved smoothness on the performance of surface acoustic wave devices is being investigated.

**Fig 11:**
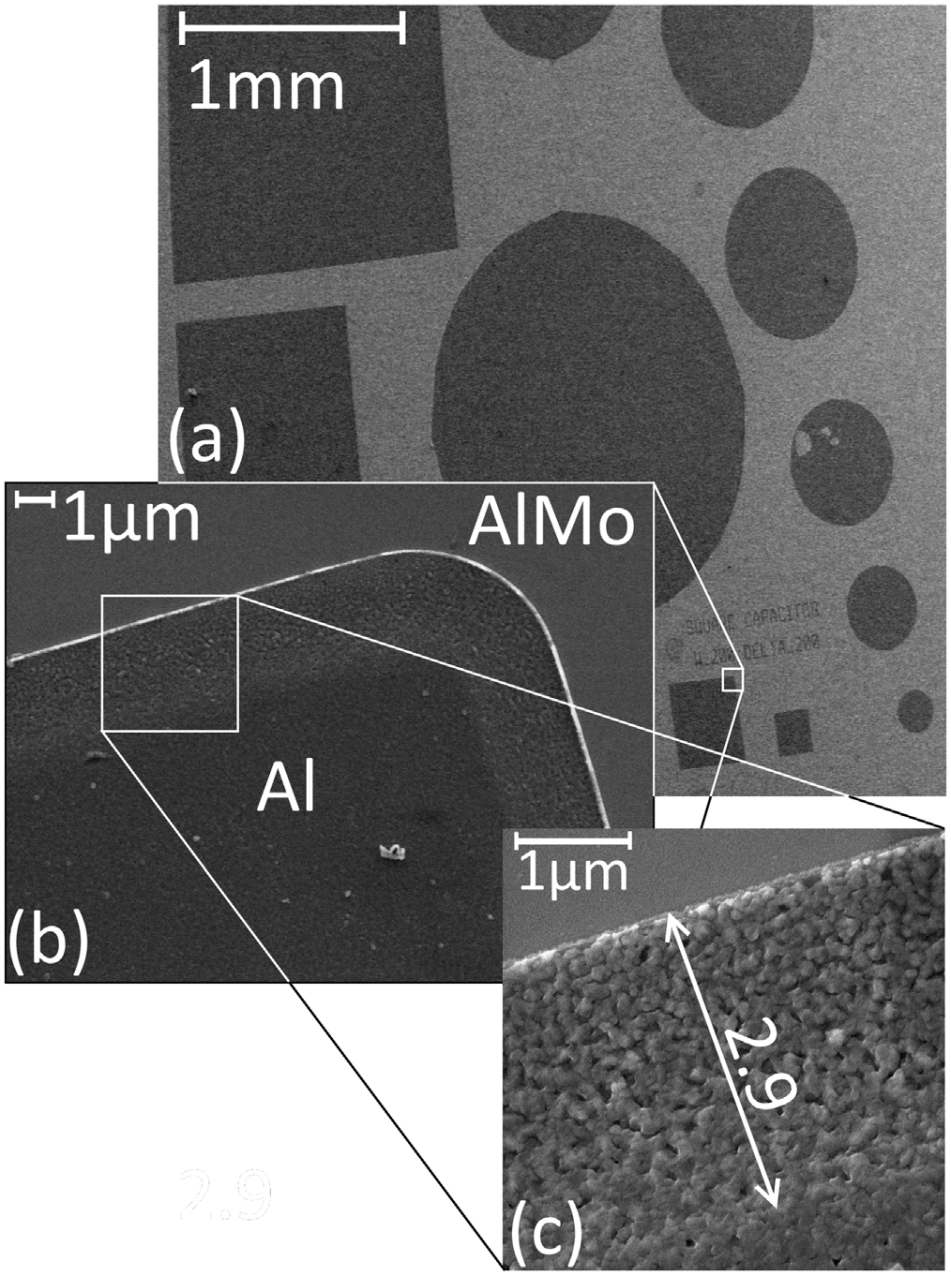
SEM images of Al-AlN-Al/0.32Mo capacitors fabricated using a lift-off process. (a) Overall picture showing square and circular capacitors with different sizes, (b) Close-up image of an edge of one of the capacitors showing the surfaces of the two different materials. (c) A higher magnification image showing the width of the thickness variation region caused by the lift-off process and the difference in roughness between Al and AlMo surfaces.

**Fig 12:**
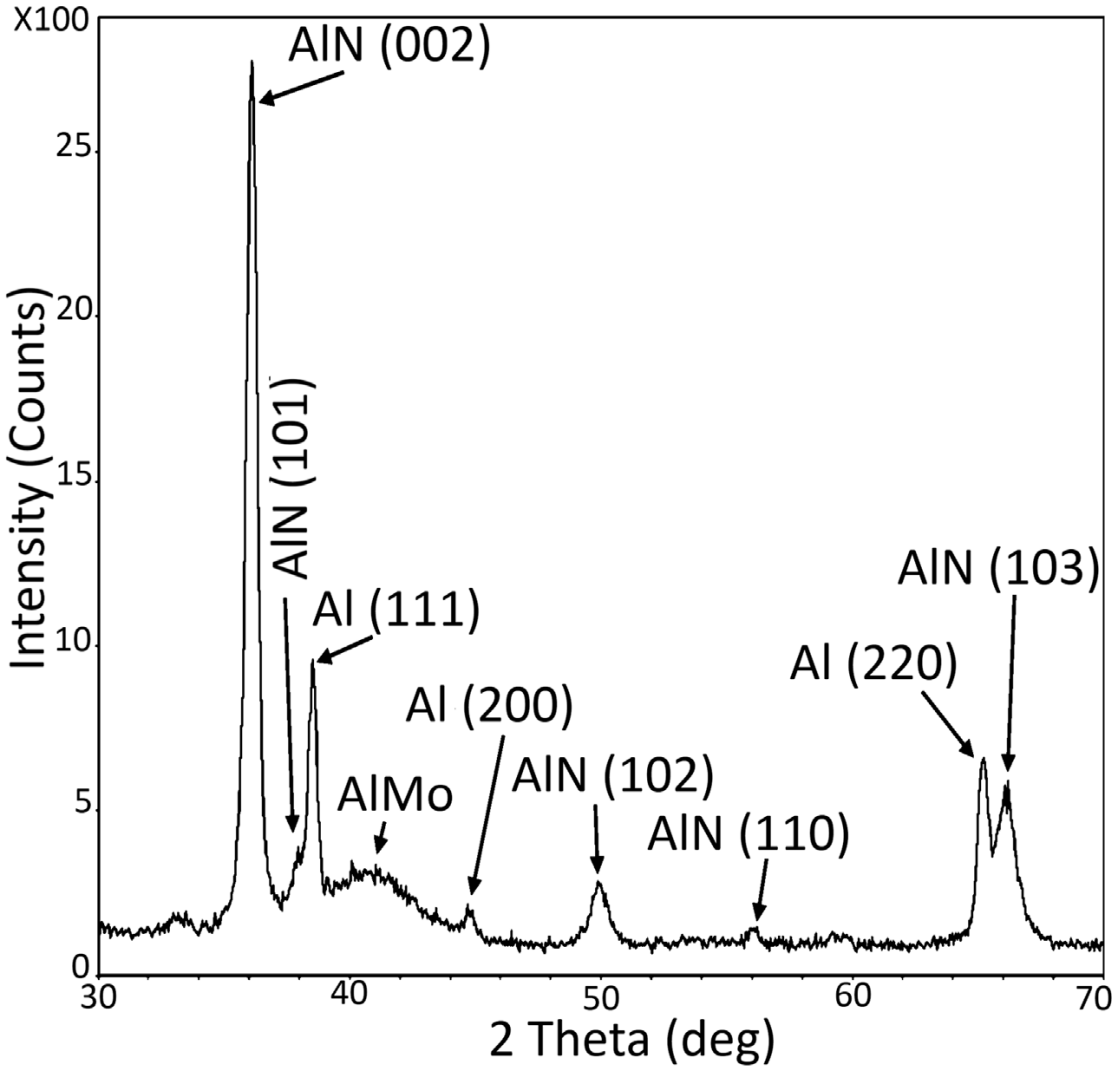
X-ray diffraction spectrum of the patterned Al-AlN-Al/0.32Mo capacitors; the spectrum illustrates the use of the lift-off process without degrading the (002) crystal growth of the AlN.

Use of a low-temperature process additionally brings about advantaged with respect to process compatibility. To demonstrate the advantage of low temperature deposition, a lift-off process of AlN c-axis oriented thin films was successfully carried out. Square and circular two plate capacitors of diameters ranging from 200μm to 1.4mm were fabricated. Scanning electron microscopy was used to investigate the resulting capacitors. Fig 11a shows an image of the various capacitors. A close-up image of the edge of one structure shows a variation in thickness across a ~3μm width. This thickness variation expected from the bi-layer lift-off process given the undercut area in the LOR-B resist. This large variation could be reduced by a better control of the undercut, or using a single resist for lift-off [36, 48]. XRD analysis was also carried out in the patterned capacitors to ensure the (002) preferential orientation of the AlN film. The XRD spectrum shown in Fig 12 depicts peaks related to the 3 layers involved (AlMo/AlN/Al). The result indicated a high (002) preferential orientation thanks to the low temperature deposition process.

**Fig 13:**
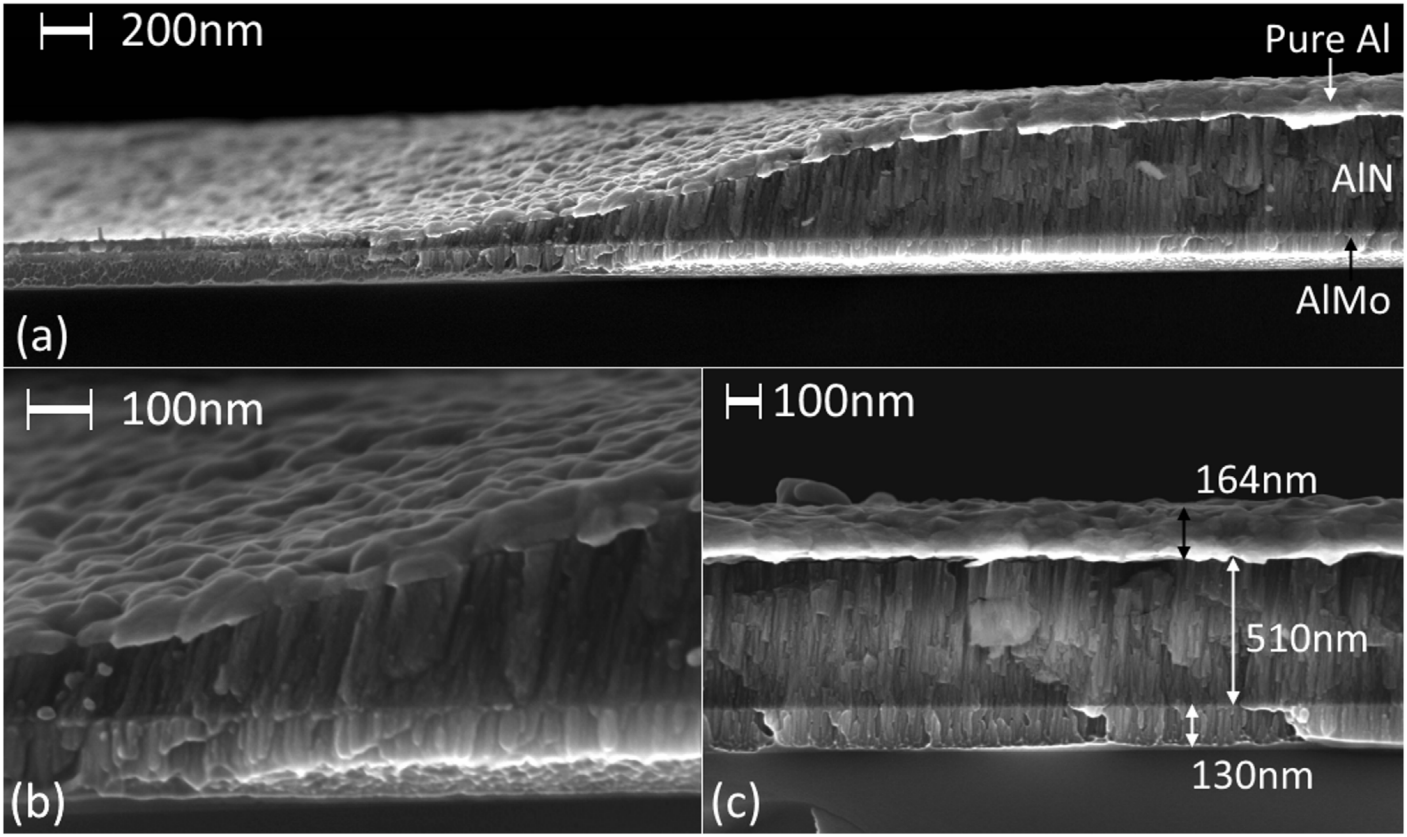
SEM images of the cross-section of the fabricated Al-AlN-Al/0.32Mo capacitors. (a) overall image of the thickness variation area, (b) a close-up image of the AlN of thickness below 250nm, (c) a close-up image of the cross section of the capacitor at a uniform thickness area.

The cross-section view of the patterned capacitor edge (Fig 13) distinctively show the three layers involved. Interestingly, columnar growth can be observed in the ultrathin layer of AlN even at thicknesses below 100nm. The columns are however grown with a slightly tilted angle (Fig 13b) due to the transportation of atoms through the undercut area of the bi-layer resist. Lifting-off ultrathin films with a single resist layer can lead to growing vertical columnar crystallites even at lower thicknesses. This can be seen from the vertical columns observed close to the AlMo/AlN interface in Fig 13c and Fig 9d compared to the disoriented columns observed at the Al/AlN interface in Fig 9c. This demonstrates the potential of using AlN-Al/0.32Mo composite layers for the realization piezoelectric nanoscale devices.

Characterization of the dielectric properties of the AlN films was carried out on the fabricated capacitors using Keithly 4200-SCS parameter analyzer. To measure the dielectric constant, the capacitance of 48 different capacitors of different shapes and sizes have been measured at 10 KHz for each deposition runs. The average value of the AlN thickness across the wafer is used to calculate the dielectric constant. We measured the dielectric constant for AlN capacitors fabricated on Al/0.32Mo and aluminum seed layers at two different sputtering powers of 200W and 300W and pressure of 1mTorr. The dielectric constant of the AlN was found to be 8.9±0.7 for Al/0.32Mo seed layer and 8.7±0.7 for aluminum, thus equivalent within experimental error.

## Conclusions

We reported a study of the deposition of c-axis oriented aluminum nitride thin films on Al/0.32Mo metallic nanocomposites at room temperature. The advantages of Al/0.32Mo are the higher tensile strength and the low surface roughness compared with pure aluminum, which shows superior mechanical stability for metallic NEMS devices. Generally, the higher the kinetic energy of the ejected aluminum atoms when they reach the substrate surface, the better c-axis crystallographic texture of the aluminum nitride thin film obtained. This explains the inverse proportionality of crystallographic texture with sputtering pressure and its direct proportionality with the sputtering power and temperature. In addition, use of a metallic glass as a seed layer yielded the deposition of AlN with piezoelectric response comparable to those reported in literature. In return, use of metallic glass as a seed layer yielded AlN films with fourfold improvement of surface roughness, an important figure of merit for applications involving surface waves and/or requiring ultra-thin layers. The impact of such improved smoothness on the performance of surface acoustic wave devices is being investigated.

The advantage of low temperature deposition of AlN was demonstrated in a simple lift-off process for thin film patterning. By integrating AlN with Al/0.32Mo in nanoscale piezoelectric bimorph structures, self-sensing NEMS devices can be realized with potential enhancement in the piezoelectric response of the AlN material.
